# Insight into Physicochemical, Rheological, and Antibacterial Properties of Chitosan Extracted from *Antarctic krill*: A Comparative Study

**DOI:** 10.3390/molecules25184074

**Published:** 2020-09-07

**Authors:** Yuan Yuan, Luzhu Chen, Wenzheng Shi, Zhihe Wang, Hongcai Zhang

**Affiliations:** 1 College of Food Science and Technology, Shanghai Ocean University, Shanghai 201306, China; yyuan@shou.edu.cn (Y.Y.); lzchen@shou.edu.cn (L.C.); wzshi1@shou.edu.cn (W.S.); 2 National R&D Branch Center for Freshwater Aquatic Products Processing Technology (Shanghai), Shanghai 201306, China; 3 School of Agriculture and Biology, Shanghai Jiao Tong University, Shanghai 200240, China; 4 State Key Laboratory for Pollution Control, School of Environmental Science and Engineering, Tongji University, Shanghai 200092, China

**Keywords:** *Antarctic krill*, crustacean wastes, chitosan, rheology, antimicrobial activity

## Abstract

In this work, physicochemical, rheological, and antibacterial properties of chitosan (CS) extracted from white shrimp (WS), giant river prawn (GP), and *Antarctic krill* (AK) were investigated. The results demonstrated that molecular weight (MW) of commercial chitosan (CCS), WSCS, GPCS, and AKCS were 1175.8, 2130.4, 1293.3, and 1109.3 kDa with the degree of deacetylation (DDA) of 73.5, 74.1, 82.1, and 75.9%, respectively. Fourier transform infrared (FT-IR), X-ray diffraction (XRD), differential scanning calorimetry (DSC), and scanning electron microscope (SEM) were employed to study the structural differences of CS. Moreover, storage modulus (G′) and loss modulus (G″) of AKCS were lower than that of WSCS and GPCS, respectively, but higher than that of CCS. Minimum inhibitory concentration (MIC) and minimum bacterial concentration (MBC) of CS against *Escherichia coli* and *Staphylococcus aureus* were investigated at concentration between 0.0125 and 1 mg/mL. These results highlighted that AKCS with low viscoelastic properties had a potential application in food and pharmaceutical application.

## 1. Introduction

With the development of aquaculture in recent years, approximately 6–8 million tons of crustacean wastes globally were produced worldwide every year [1]. It consists of 30–40% of proteins, 30–50% of CaCO_3_ and Ca_3_(PO_4_)_2_, and 20–30% of chitin [2]. However, crustacean wastes are abundantly discarded, leading to critical environmental pollution, as well as the wastes of valuable biomaterials. *Antarctic krill* (AK), as a key species in the Antarctic ecosystem [3], with the biomass of 342–356 million tons with high-quality useful components, are mostly used for aquatic feed with low commercial value [4]. Previous study has reported that AK contained 77.9–83.1% moisture, 11.9–15.4% proteins, 0.4–2.6% lipids, and approximately 2% chitin [5]. Among them, protein and lipid have been commercially developed, and discarded AK shells could be utilized as a valuable source of chitosan (CS) [6].

CS, a deacetylated derivative of chitin, is recognized as one of the most abundant polysaccharides occurring in nature next to cellulose [7,8]. CS extracted from crustacean shell, squid, mushrooms, coral eggs, etc. could be widely used in several applications due to its excellent properties including biodegradability, biocompatibility, non-toxicity, and chelating ability of metal ions [9,10]. Three steps including deproteinization, demineralization, and removal of lipids and pigments were employed to prepare the chitin firstly [11]. Then, the conversion of chitin into CS was mostly conducted by strong alkaline deacetylation of chitin due to high efficiency. The preparation methods of CS using shrimp, crab, fishery, etc. has been reported in previous works [12,13,14]. Based on our best knowledge, no study has reported the preparation of AKCS where other crustaceans were also considered in detail.

In this work, CS was extracted from white shrimp (WS), giant river prawn (GP), and AK for comparing its physicochemical, structural, rheological, and antimicrobial properties. This study would provide a detailed exploration of CS extracted from crustacean shells, which was particularly beneficial to scale up their application in food and pharmaceuticals fields.

## 2. Materials and Methods

### 2.1. Materials and Reagents

WS and GP were purchased from No.157, Guzong Road, Nonggongshang supermarket in Shanghai, China. AK were caught in FAO 48.2 fishing area in March 2012, frozen and stored at −20 °C. Commercial CS, NaOH (96%), HCl (38%), K_2_MnO_4_ (99.5%), oxalic acid (99.5%), C_2_H_5_OH (99.5%), nutrient agar, and trypticase soy broth (TSB) were obtained from Sinopharm Chemical Reagent Co. Ltd. (Shanghai, China).

### 2.2. Preparation of WSCS, GPCS, and AKCS

Three kinds of crustacean shells were thoroughly washed with deionized water and dried in an oven at 60 °C overnight until a constant weight. The dried samples were completely smashed using ultrafine grinder (Cerendiptor MKCA 63, Masuko, Japan) and filtered with 200 mesh number sieve.

The accurately weighed samples were treated with 40% NaOH (*w*/*w*) at 100 °C for 1 h to remove proteins, and then soaked in 5% HCl (*w*/*w*) solution with rotation at 750 rpm for 24 h at ambient temperature to remove CaCO_3_ and other minerals. Obtained chitin was decolored using 10% (*v*/*v*) H_2_O_2_ at 100 °C for 5 h, followed by washing using DI water and freeze-dried (LABCONCO 7,948,030 Co., USA) [11]. Finally, 2% K_2_MnO_4_ and 2% oxalic acid were employed at 60 °C for 1 h for the removal of pigments of chitin. Chitin was then deacetylated using 40% (*w*/*v*) NaOH. The mixtures were incubated in a 100 °C water bath for 1 h with constant stirring at 750 rpm and then deacetylated again under the same conditions. The remaining CS solids were filtered and freeze-dried after adjusting a final pH 11 using 4 N NaOH [10].

### 2.3. Physicochemical Properties of Prepared CS

#### 2.3.1. Yield and Solubility

Accurately weighted CS was dried in a vacuum drying oven at 30 °C overnight until a constant weight for calculating its yields.

(1) $$\mathrm{Yield}\;(\%)=\frac{\mathrm{Weight}\;\mathrm{of}\;\mathrm{chitosan}\;\mathrm{after}\;\mathrm{drying}}{\mathrm{Weight}\;\mathrm{of}\;\mathrm{added}\;\mathrm{crustacean}\;\mathrm{shells}\;}\times 100\%$$

The 0.1 g of CS was weighted and dissolved in 10 mL of 1% acetic acid at 30 °C before centrifugation for 30 min [15]. The supernatant was removed and the sediment was dried at 60 °C overnight until a constant weight.

(2) $$\mathrm{Solubility}\;(\%)=\frac{\mathrm{Soluble}\;\mathrm{chitosan}\;\mathrm{weight}}{\mathrm{Added}\;\mathrm{chitosan}\;\mathrm{weight}}\times 100\%$$

#### 2.3.2. Ash and Moisture Content

The 1 g of CS was dried in a vacuum drying oven at 80 °C for 4 h after carbonization for 2 h until constant weight. The 0.5 g of CS samples were dried at 105 °C for 2 h until a constant weight. Moisture content of CS was calculated as follows.

(3) $$\mathrm{Moisture}\;\mathrm{content}\;(\%)=\frac{\mathrm{Wet}\;\mathrm{weight}\;-\mathrm{dry}\;\mathrm{weight}}{\mathrm{Wet}\;\mathrm{weight}}\times 100\%$$

#### 2.3.3. Viscosity Average Molecular Weight (MW) and Degree of Deacetylation (DDA)

Accurately weighed CS was dissolved in 0.5 M acetic acid and 0.5 M sodium acetate buffer at 25 °C for the determination of intrinsic viscosity [η]. MW of CS was estimated using Mark-Houwink-Sakurada equation [16].

(4) $$\left[\eta \right]=K\;\left[M\nu \right]{\;}^{a}$$

where K = 1.81 × 10^−3^ and a = 0.93 are constants in Mark-Houwink-Sakurada equation.

The 0.1 g of CS was completely dissolved in 25 mL of HCl standard solution (0.1 M) with help of ultrasound treatment. Then, CS solution was titrated with 0.1 M NaOH standard solution until the appearance from blue to green color using methyl red aniline blue as indicator. DDA of CS was calculated as followed [17]:

(5) $${\mathrm{NH}}_{2}\;(\%)=\frac{(C_{1}V_{1}-C_{2}V_{2})\times 0.016}{G(100-W)}\times 100$$

(6) $$\mathrm{DDA}(\%)=\frac{{NH}_{2}}{9.94}\times 100$$

where *C*_1_ and *C*_2_ are the concentration of HCl standard solution (0.1008 mol/L) and NaOH standard solution, respectively. *V*_1_ and *V*_2_ are volume of HCl standard solution and consumed NaOH solution, respectively. The 9.94 is the theoretical amino content. G and W are weight and moisture content of CS, respectively.

### 2.4. Scanning Electron Microscopy (SEM) Analysis of WSCS, GPCS, and AKCS

SEM (Hitachi S-3400N, Tokyo, Japan) was used to observe the surface morphologies of WSCS, GPCS, and AKCS. The surface of CS was spread with a layer of gold before test. Surface images were taken at an acceleration voltage of 20 kV and magnified to 20 µm.

### 2.5. Structural Properties of WSCS, GPCS, and AKCS

#### 2.5.1. Fourier Transform Infrared (FT-IR) Analysis

Samples were blended with KBr (1:99, *w*/*w*) and analyzed by FT-IR analyzer (Perkin Elmer Spectrum RXI, Naperville, IL, USA). Spectral scanning was taken in the wavelength regions between 4000 and 400 cm^−1^ at a resolution of 4 cm^−1^, and all samples were recorded at 16 scans. A background scanning was conducted before measurement and subtracted from sample spectra.

#### 2.5.2. Differential Scanning Calorimetry (DSC) Analysis

Accurately weighed 5 mg CS was placed into aluminum cup and left for equilibration at 2 °C for 2 h, and empty aluminum cup was used as reference. All samples were heated to 500 °C using. DSC analyzer (model SDT Q600 Netzsch, Selb, Germany) at a heating rate of 10 °C/min and a gas flow of 23 mL/min. After ramp, a cooling ramp was started with a rate of 10 °C/min down to 2 °C and left for equilibration for 2 h.

#### 2.5.3. X-ray Diffraction (XRD) Analysis

XRD analysis was recorded at a scan rate of 10°/min with the scan angle from 5 to 80°using D8 advance (Bruker, Karlsruhe, German) (30 kV and 40 mA). Crystallinity index (*C*rI) was calculated using the following formula [18]:

(7) $$C\mathrm{rI}\;(\%)=\frac{I_{100}-I_{am}}{I_{100}}\times 100\%$$

where I_110_ is the maximum intensity at 20° and *I_am_* is the maximum intensity of amorphous diffraction at 16°.

### 2.6. Rheological Properties of WSCS, GPCS, and AKCS

CS solution (0.5%) was completely dissolved in 1% aqueous acetic acid (*w*/*v*) and configured to reduce impurities and air bubbles at 12,000 rpm for 5 min. Rheological properties of CS were measured from 0.01 to 1000 1/s at 20 °C using rheometer (MCR301 Anton paar, Shanghai, China). To further investigate the storage modulus (G′) and loss modulus (G”), a frequency sweep was determined from 0.1 to 5 Hz with the strain of 2% at 20 °C. Parameters of G′ and G′′ as a function of angular frequency were directly obtained from computer software (Rheological Advantage Data Analysis Program, TA Instruments, New Castle, DE, USA).

Experimental data were fitted by power-law equations:

(8) $$G^{\prime }=K^{\prime }\omega^{n\prime }$$

(9) $$G^{\prime \prime }=K^{\prime \prime }\omega^{n^{\prime \prime }}$$

where *G′* is storage modulus (Pa), *G*” is loss modulus (Pa), *K′*, *K*”, $\omega^{n^{\prime }}$, and $\omega^{n^{\prime \prime }}$ are constants [19].

For steady shear measurements, shear rates ranging from 1 to 100 s^−1^ were used with shear time of 2 min. The relationship between shear stress and shear rate was recorded at 25 °C. Experimental data were fitted by a Herschel-Bulkley’s model [19]:

(10) $$\tau =\tau_{0}+K\varepsilon^{n}$$

where s is shear stress (Pa), τ_0_ is yield stress (Pa), K is consistency coefficient, ε is shear rate (s^−1^), and *n* is flow behavior index.

### 2.7. Antimicrobial Activity of WSCS, GPCS, and AKCS

Minimum inhibitory concentration (MIC) and minimum bacterial concentration (MBC) were used to measure the antibacterial activity of CS. MIC was determined by a turbidimetric method based on previous report [20]. The 1, 0.5, 0.25, 0.125, and 0.0625 mg/mL of CS solutions were prepared by double-dilution method. *Escherichia coli* (G^−^) and *Staphylococcus aureus* (G^+^) were cultured in TSB medium and diluted to about 10^7^ CFU.

All sample solutions were accurately quantified and added into DI water. For the first tube, 5.0 mL CS solutions (1 mg/mL) was added, respectively. After mixing, 5.0 mL of mixtures was transferred to the second tube, and similar transformation was repeated. Hence, each tube contained a test sample solution with half of the concentration of the previous one. The tubes were inoculated with 50 μL of freshly prepared bacteria suspension under aseptic conditions. All samples were adjusted to pH 4.5 with 0.1 M NaOH for equal comparisons and incubated at 35 °C for 24 h to evaluate MIC subsequently. MBC is defined as the concentration producing a 99.9% reduction of colony number in the initial inoculum by assaying the live organisms in those tubes from the MIC test that showed no growth.

### 2.8. Data Analysis

One-way ANOVA analysis was carried out to determine the significant differences using the SPSS program (SPSS 24.0, IBM SPSS Institute, Inc., Chicago, IL, USA). The pictures were processed using Origin 9.0 (Origin Lab Inc., Northampton, MA, USA). Data differences were considered to be statistically significant when *p* < 0.05. All data were conducted at least in triplicate and reported as mean values ± standard errors.

## 3. Results and Discussion

### 3.1. Physicochemical Analysis of WSCS, GPCS, and AKCS

#### 3.1.1. Yields and Solubility

**Figure 1:**
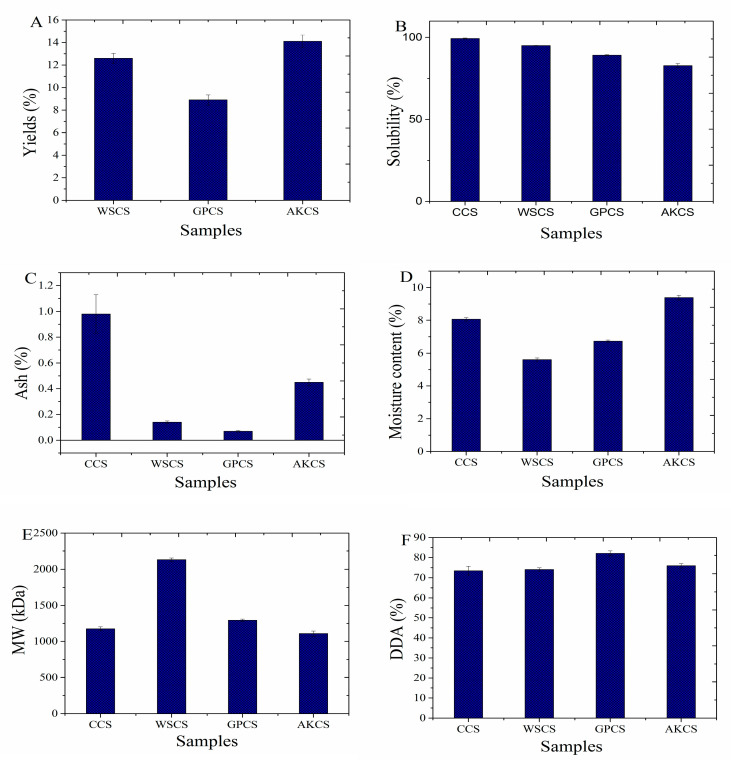
Yield (**A**), solubility (**B**), ash (**C**), moisture content (**D**), MW (**E**), and degree of deacetylation (DDA) (**F**) of CCS, WSCS, GPCS, and AKCS. CCS: commercial chitosan; WSCS: white shrimp chitosan; GPCS: giant river prawn chitosan; AKCS: *Antarctic krill* chitosan; MW: molecular weight.

As shown in Figure 1, yields of WSCS, GPCS, and AKCS were 12.6 ± 0.44, 8.9 ± 0.46, and 14.1 ± 0.56%, respectively. The results indicated that AK was a desirable CS source. Solubility of CCS, WSCS, GPCS, and AKCS was 99.3 ± 0.26, 95.0 ± 0.15, 89.2 ± 0.32, and 82.8 ± 1.29%, respectively (Figure 1), indicating that CC has the highest solubility. Solubility of CCS mainly depended on the reaction temperature during deacetylation [21]. Hossain and Iqbal [22] found that the solubility of CS could be affected by several critical factors including deacetylation reaction time, temperature, alkali concentration, and particle size. Moreover, solubility of CS could also be affected by MW and biological origins of CS [23].

#### 3.1.2. Ash and Moisture Content

Ash contents of CCS, WSCS, GPCS, and AKCS were 0.98 ± 0.01, 0.14 ± 0.01, 0.07 ± 0.006, and 0.45 ± 0.02%, respectively. It was indicated that GPCS has the lowest ash content, which was related to the DDA and origins of CS. Ash content of CCS is an important parameter affecting its solubility, viscosity, and other physicochemical characteristics [12]. Moisture of CCS, WSCS, GPCS, and AKCS reached 8.06 ± 0.11, 5.61 ± 0.10, 6.73 ± 0.073, and 9.39 ± 0.15%, respectively (Figure 1). Moisture contents were also one of the significant properties to evaluate the characteristics of CS because water molecules could be adsorbed on the polymeric chains and acted as a plasticizer effect on thermal stability of CS [12]. Moreover, moisture contents of CS were also closely related to the purity of CS influenced by its origins [23].

#### 3.1.3. MW and DDA

MW of CCS, WSCS, GPCS, and AKCS was 1175.8 ± 27.31, 2130.4 ± 23.35, 1293.3 ± 17.16, and 1109.3 ± 35.62 kDa, respectively (Figure 1). Previous studies reported that low MW CS had better antiseptic [24] and anticancer [25] ability than higher MW one. The main factors of influencing MW of CS consisted of extraction time, temperature, reagent concentration of CS, etc. [26]. In addition, Younes also indicated that MW of CS varied with its origins and the residual aggregates in the matrix [27].

DDA of CCS, WSCS, GPCS, and AKCS was 73.5 ± 2.13, 74.1 ± 0.85, 82.1 ± 1.28, and 75.9 ± 1.07%, respectively. It is well-known that DDA influences the physical, chemical, and biological properties of CS including electrostatic characteristics, biodegradability, self-aggregation, adsorption properties, and chelating ability of metal ions [28]. DDA, representing the removal of acetyl group of chitin, mainly determines the content of free amino groups in the polysaccharides, which was affected by crustacean species and preparation process [29]. Moreover, alkali concentration was also recognized as an important factor to influence DDA value [30]. During DDA, preferred 60% of NaOH (*w*/*w*) concentration and 12 h of reaction time were the optimal reaction conditions for removing the acetyl groups according to previous reports [31,32].

### 3.2. SEM Observation

**Figure 2:**
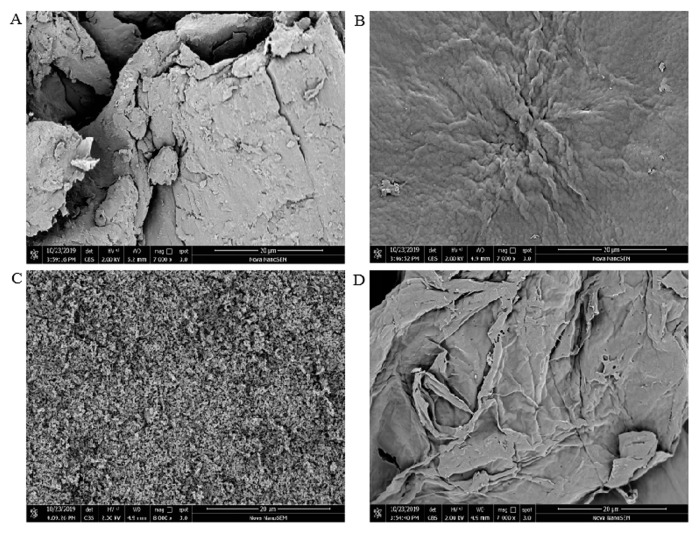
SEM images (20 μm) of CCS (**A**), WSCS (**B**), GPCS (**C**), and AKCS (**D**). CCS: commercial chitosan; WSCS: white shrimp chitosan; GPCS: giant river prawn chitosan; AKCS: *Antarctic krill* chitosan.

SEM images of CCS, WSCS, GPCS, and AKCS showed in Figure 2 had obvious differences. WSCS and AKCS showed rougher surface morphology and ridges than CCS and GPCS (Figure 2B,D). Moreover, AKCS displayed smooth microfibrillar crystalline structure and layer structure largely intact (Figure 2D). It was found that surface structure of WSCS was more compact than that of AKCS. The micrograph of GPCS showed granular bulge and porosity structure in Figure 2C, but smooth and flat surface for CCS (Figure 2A). It could be explained that the four kind CS has different origins.

### 3.3. Structural Analysis of CCS, WSCS, GPCS, and AKCS

#### 3.3.1. FT-IR Analysis

**Figure 3:**
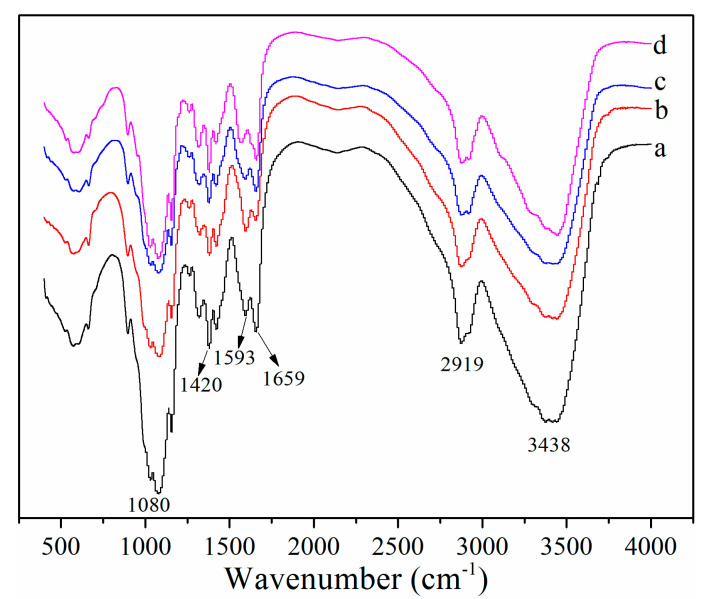
FT-IR analysis of CCS (**a**), WSCS (**b**), GPCS (**c**), and AKCS (**d**). CCS: commercial chitosan; WSCS: white shrimp chitosan; GPCS: giant river prawn chitosan; AKCS: *Antarctic krill* chitosan.

Peaks of CS from crustaceans shells at 3438, 2919, 1659, 1593, 1420, and 1080 cm^−1^ represented -OH, -CH, amide I, NH_2_ bending vibration, -OH and -CH, and -C-O stretching vibrations, respectively (Figure 3) [11,33,34]. Furthermore, the specific bands of -C-O and -C-O-C stretching absorbing between 1052 and 1030 cm^−^^1^ were observed for all CS. All CS showed the similar FT-IR curves, and the absorbance of peaks around 1593 cm^−1^ represented the -NH_2_ bending vibration in amino group, which indicated the difference of DDA among CS [35]; the higher the peak was, the higher DDA was, which was consistent with DDA results (Figure 1).

#### 3.3.2. DSC Analysis

**Figure 4:**
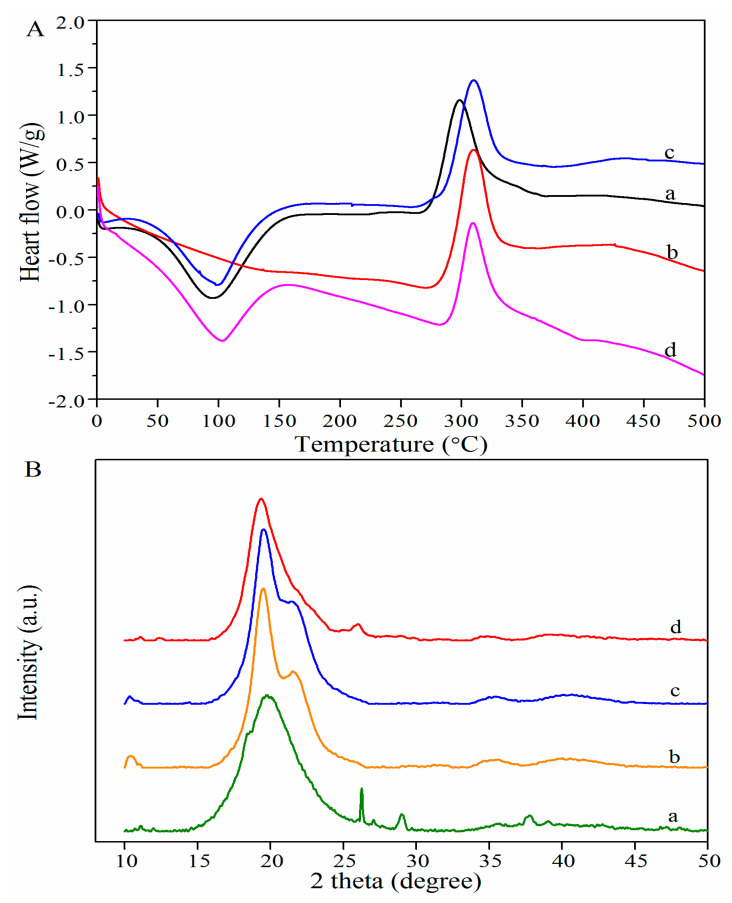
DSC (**A**) and XRD (**B**) analysis of CCS (**a**), WSCS (**b**), GPCS (**c**), and AKCS (**d**). CCS: commercial chitosan; WSCS: white shrimp chitosan; GPCS: giant river prawn chitosan; AKCS: *Antarctic krill* chitosan.

DSC curves of CCS, WSCS, GPCS, and AKCS are presented in Figure 4A. All samples showed the endothermic peak at 100–110 °C, attributing to the evaporation of absorbed and bound water [36]. The reason why WSCS did not display the endothermic peak was probably due to the lower moisture content showed in Figure 1. All samples had similar exothermic peak appearing around 300–310 °C due to the glass transition followed by decomposition [37]; among them, the exothermic peak maximum of GPCS was slightly shifted rightwards, it might be explained that moisture (entrapped inside CS structure) evaporation from amorphous structure of GPCS, as well as the availability of less polar -NH_2_ groups [38].

Thermoanalytic parameters obtained from DSC curves are summarized in Table 1. The onset temperatures (T_o_) were in the range of 276.75–2293.06 °C with the descending order of GPCS ≈ AKCS < WSCS < CCS. Thus, GPCS and AKCS exhibited higher thermal stability than that of WSCS and CCS. The slight discrepancy in the peak temperatures (T_p_) might be due to the diversity of CS origins and the different preparation methods applied. The completion temperatures (T_c_) were in the range of 353.27–371.54 °C with the descending order of CCS < WSCS < GPCS < AKCS. The decomposition enthalpies (∆H) of CCS, WSCS, GPCS, and AKCS were 235.07, 193.00, 154.09, and 145.08 J/g, respectively [39]. The higher the ∆H was, the lower the thermal stability was, indicating GPCS has the lowest thermal stability [14].

#### 3.3.3. XRD Analysis

XRD spectra of CS present characteristic peaks at 20°, which was in agreement with previous reports (Figure 4B). Jung, Kuk, Kim, and Park also reported the one characteristic crystalline reflection at 19.9° for crustacean CS [40]. CCS has several small reflection peaks compared to others because of high ash content [10]. *C*rI of CS mainly depended on several factors including origins, preparation conditions, DDA, etc. *C*rI of CCS, WSCS, GPCS, and AKCS reached 89.86, 83.25, 84.27, and 90.39%, respectively (Table 2), indicating AKCS with high *C*rI was related to crystal II in CS structure [41,42].

### 3.4. Rheological Measurements of CCS, WSCS, GPCS, and AKCS

**Figure 5:**
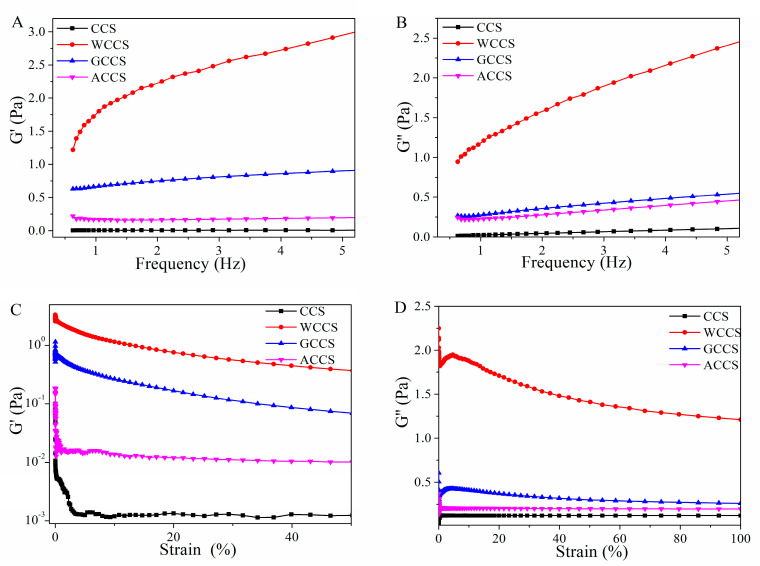
Storage modulus G′ (**A**,**C**) and loss modulus G” (**B**,**D**) of CCS, WSCS, GPCS, and AKCS vs. frequency (strain). CCS: commercial chitosan; WSCS: white shrimp chitosan; GPCS: giant river prawn chitosan; AKCS: *Antarctic krill* chitosan.

A strain sweep was carried out at a constant frequency (1 Hz) under 20 °C to investigate the relationship between modulus and strain or frequency. The G′ and G” are demonstrated in Figure 5A,B, respectively. Both G′ and G” values of WSCS gradually increased in a frequency range from 0.1 to 5 Hz for forming a more compact matrix structure. The G′ value was greater than G”, leading to low mechanical strength and high viscosity strength [43,44]. The G′ and G” of WCCS were higher than other CS, which indicated the higher viscosity and more stability than others. The order of G′ and G” were the ascending order of WCCS > GPCS > AKCS > CCS [44]. Moreover, G′ value decreased whereas the viscous character increased (G′′), avoiding the crossover of the moduli throughout the frequency range studied.

**Figure 6:**
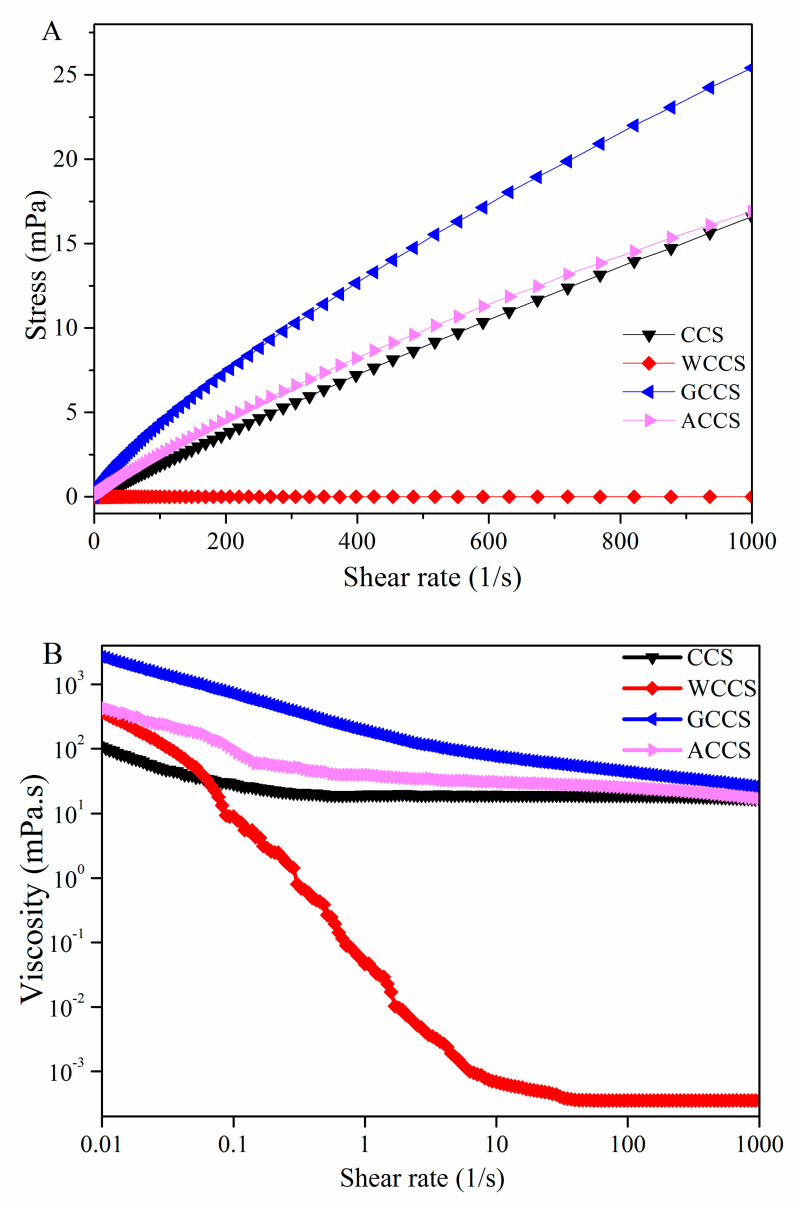
Stress (**A**) and viscosity (**B**) vs. shear rate (1/s) of CCS, WSCS, GPCS, and AKCS solutions. CCS: commercial chitosan; WSCS: white shrimp chitosan; GPCS: giant river prawn chitosan; AKCS: *Antarctic krill* chitosan.

The apparent viscosity of non-Newtonian fluids decreased with increasing shear rate in a range of 0.001–1000 s^−1^ at 20 °C (Figure 6). The graph indicated a drastic decrease in viscosity of CS at the beginning, validating the shear-thinning behavior and pseudo-plastic fluid of CS. GPCS with linear molecules and rigidity showed the most stable viscosity [45], followed by AKCS, CCS, and WCCS. It was found that viscosity of CS might be due to intertwining of polymer chains, which revealed the entanglement density affected by MW and resulted in a decrease of apparent viscosity [46]. In addition, the viscosity of CS was also influenced by its conformation and structural flexibility and linear molecules [47].

### 3.5. Antimicrobial Activity

**Figure 7:**
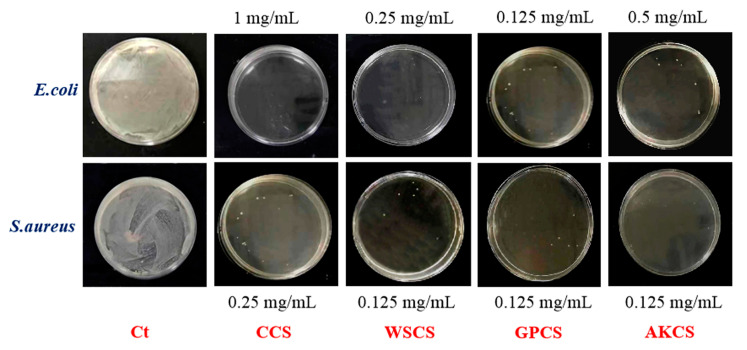
Colony growth of CCS, WSCS, GPCS, and AKCS solutions for MBC. Ct: controls; CCS: commercial chitosan; WSCS: white shrimp chitosan; GPCS: giant river prawn chitosan; AKCS: *Antarctic krill* chitosan.

MIC and MBC of CCS, WSCS, GPCS, and AKCS against *E. coli* and *S. aureus* are presented in Table 2. Colony growth of CCS, WSCS, GPCS, and AKCS solutions for MBC measurement is shown in Figure 7. The order of MIC ranking for *E. coli* was GC < WS < AC < CC, and GC < WS ≈ AC < CC for *S. aureus*. GPCS has the lowest MIC (0.0625 mg/mL) and MBC (0.125 mg/mL), indicating its superior antibacterial activities against *E. coli* and *S. aureus*. The reason why GPCS exhibited the higher MIC and MBC was because of its high DDA and low MW [48], which was consistent with physicochemical parameters (Figure 1). WSCS, GPCS, and AKCS had the same MBC, lower than CCC against *S. aureus*. The antimicrobial activity of CS affected by several factors including MW, DDA%, microorganism species, ionic strength, and pH of matrix [49]. In general, antibacterial activity of CS was more effective against *E. coli* than *S. aureus*, which was in accordance with previous studies [50,51].

Antibacterial mechanisms of CS have been reported in several published literatures. (1) Cationic chains of CS polymer are attached to the outer membrane of G^+^ bacteria through more effective electrostatic interaction than G^-^ did, thus rendering the excellent antibacterial activity, (2) CS could penetrate the bacterial cell wall, inhibiting the synthesis of mRNA and DNA transcription [52], (3) CS could play a key role on the uptake and secretion of substrates for inhibiting enzyme activity and killing the bacteria [53,54].

## 4. Conclusions

In this work, CS was extracted from WS, GP, and AK for investigating its physicochemical, rheological, and antibacterial properties. DDA of *CCS, WSCS, GPCS, and AKCS* were 73.5, 74.1, 82.1, and 75.9% with MW of 1175.8, 2130.4, 1293.3, and 1109.3 kDa, respectively. Compared with WSCS and GPCS, AKCS had low G′ and G”, but poor antibacterial activity. All CS exhibited the viscoelastic properties, among them, WSCS had higher viscosity and more stability than others based on the results of rheological measurements. MIC and MBC results indicated that GPCS presented the best bacterial activity against *E. coli* and *S. aureus*. This study will provide a new idea for expanding the utilization of AK and other crustacean wastes in the near further.

## Figures and Tables

**Table 1:** Differential scanning calorimetry (DSC) parameters of CCS, WSCS, GPCS, and AKCS.

Samples	DSC				XRD
	T_o_ (°C) ^++^	T_p_ (°C)	T_c_ (°C)	∆H (J/g)	*C*rI (%)
CCS ^+^	276.75 ± 6.57 ^a^^+++^	298.39 ± 4.68 ^a^	371.54 ± 10.24 ^a,b^	235.07 ± 5.38 ^c^	89.86 ± 1.31 ^b^
WSCS	288.20 ± 6.45 ^a^	309.60 ± 5.32 ^a^	359.62 ± 8.35 ^a^	193.00 ± 6.24 ^b^	83.25 ± 1.54 ^a^
GPCS	293.06 ± 8.24 ^a^	309.96 ± 7.38 ^a^	355.24 ± 9.15 ^a^	154.09 ± 7.05 ^a,b^	84.27 ± 1.86 ^a^
AKCS	292.51 ± 7.39 ^a^	309.14 ± 9.05 ^a^	353.27 ± 7.68 ^a^	145.08 ± 5.39 ^a^	90.39 ± 1.44 ^b^

^+^ CCS: commercial chitosan; WSCS: white shrimp chitosan; GPCS: giant river prawn chitosan; AKCS: *Antarctic krill* chitosan. ^++^ Different superscript letters (a–c) in the same column indicate a significant difference (*p* < 0.05). ^+++^ T_o_: onset temperature; T_p_: peak temperature; T_c_: completion temperature; ∆H: peak enthalpy (J/g, dry weight); *C*rI: crystallinity index.

**Table 2:** MIC and MBC of four kinds of chitosan.

Samples	MIC (mg/mL)		MBC (mg/mL)	
	*E. coli*	*S. aureus*	*coli*	*S. aureus*
CCS	0.5	0.25	1	0.25
WSCS	0.125	0.125	0.25	0.125
GPCS	0.0625	0.0625	0.125	0.125
AKCS	0.25	0.125	0.5	0.125

CCS: commercial chitosan; WSCS: white shrimp chitosan; GPCS: giant river prawn chitosan; AKC: *Antarctic krill* chitosan. MIC and MBC represented minimal inhibitory concentration and minimum bactericidal concentration, respectively.

## Abbreviations

CS: chitosan; CCS: commercial chitosan; WS: white shrimp; GP: giant river prawn; AK: *Antarctic krill*; MW: molecular weight; DDA: degree of deacetylation; *C*rI: crystallinity index; FT-IR: Fourier transform infrared; XRD: X-ray diffraction; DSC: differential scanning calorimetry; SEM: scanning electron microscope; T_o_: onset temperature; T_p_: peak temperature; T_c_: completion temperature; ∆H: peak enthalpy (J/g, dry weight); G′: storage modulus; G″: loss modulus; MIC: minimum inhibitory concentration; MBC: minimum bacterial concentration.
